# Immune-based personalized elimination diet for the treatment of irritable bowel syndrome: a double-blind randomized sham-controlled study

**DOI:** 10.1093/gastro/goag058

**Published:** 2026-06-12

**Authors:** Offir Ukashi, May Tzur, Doron Yablecovitch, Adi Lahat, Ido Laish, Ahmad Albshesh, Sandra Neuman, Limor Selinger, Orit Picard, Miri Yavzori, Rita Lipov, Ella Fudim, Revital Dvir, Alon Lang, Eyal Shachar, Uri Kopylov, Dan Carter, Shomron Ben-Horin, Tal Engel

**Affiliations:** Gastroenterology Institute, Sheba Medical Center Tel Hashomer, Ramat Gan, 56121, Israel and Faculty of Medical and Health Sciences, Tel Aviv University, Tel Aviv, 6997801, Israel; Gastroenterology Institute, Sheba Medical Center Tel Hashomer, Ramat Gan, 56121, Israel and Faculty of Medical and Health Sciences, Tel Aviv University, Tel Aviv, 6997801, Israel; Gastroenterology Institute, Sheba Medical Center Tel Hashomer, Ramat Gan, 56121, Israel and Faculty of Medical and Health Sciences, Tel Aviv University, Tel Aviv, 6997801, Israel; Gastroenterology Institute, Sheba Medical Center Tel Hashomer, Ramat Gan, 56121, Israel and Faculty of Medical and Health Sciences, Tel Aviv University, Tel Aviv, 6997801, Israel; Gastroenterology Institute, Sheba Medical Center Tel Hashomer, Ramat Gan, 56121, Israel and Faculty of Medical and Health Sciences, Tel Aviv University, Tel Aviv, 6997801, Israel; Gastroenterology Institute, Sheba Medical Center Tel Hashomer, Ramat Gan, 56121, Israel and Faculty of Medical and Health Sciences, Tel Aviv University, Tel Aviv, 6997801, Israel; Gastroenterology Institute, Sheba Medical Center Tel Hashomer, Ramat Gan, 56121, Israel and Faculty of Medical and Health Sciences, Tel Aviv University, Tel Aviv, 6997801, Israel; Gastroenterology Institute, Sheba Medical Center Tel Hashomer, Ramat Gan, 56121, Israel and Faculty of Medical and Health Sciences, Tel Aviv University, Tel Aviv, 6997801, Israel; Gastroenterology Institute, Sheba Medical Center Tel Hashomer, Ramat Gan, 56121, Israel and Faculty of Medical and Health Sciences, Tel Aviv University, Tel Aviv, 6997801, Israel; Gastroenterology Institute, Sheba Medical Center Tel Hashomer, Ramat Gan, 56121, Israel and Faculty of Medical and Health Sciences, Tel Aviv University, Tel Aviv, 6997801, Israel; Gastroenterology Institute, Sheba Medical Center Tel Hashomer, Ramat Gan, 56121, Israel and Faculty of Medical and Health Sciences, Tel Aviv University, Tel Aviv, 6997801, Israel; Gastroenterology Institute, Sheba Medical Center Tel Hashomer, Ramat Gan, 56121, Israel and Faculty of Medical and Health Sciences, Tel Aviv University, Tel Aviv, 6997801, Israel; Gastroenterology Institute, Sheba Medical Center Tel Hashomer, Ramat Gan, 56121, Israel and Faculty of Medical and Health Sciences, Tel Aviv University, Tel Aviv, 6997801, Israel; Gastroenterology Institute, Sheba Medical Center Tel Hashomer, Ramat Gan, 56121, Israel and Faculty of Medical and Health Sciences, Tel Aviv University, Tel Aviv, 6997801, Israel; Gastroenterology Institute, Sheba Medical Center Tel Hashomer, Ramat Gan, 56121, Israel and Faculty of Medical and Health Sciences, Tel Aviv University, Tel Aviv, 6997801, Israel; Gastroenterology Institute, Sheba Medical Center Tel Hashomer, Ramat Gan, 56121, Israel and Faculty of Medical and Health Sciences, Tel Aviv University, Tel Aviv, 6997801, Israel; Gastroenterology Institute, Sheba Medical Center Tel Hashomer, Ramat Gan, 56121, Israel and Faculty of Medical and Health Sciences, Tel Aviv University, Tel Aviv, 6997801, Israel; Gastroenterology Institute, Sheba Medical Center Tel Hashomer, Ramat Gan, 56121, Israel and Faculty of Medical and Health Sciences, Tel Aviv University, Tel Aviv, 6997801, Israel; Gastroenterology Institute, Sheba Medical Center Tel Hashomer, Ramat Gan, 56121, Israel and Faculty of Medical and Health Sciences, Tel Aviv University, Tel Aviv, 6997801, Israel

**Keywords:** IBS, IBS-SSS, Alcat, leukocyte activation, dietary intervention

## Abstract

**Background:**

Dietary interventions and multiple food-item restrictions are challenging for patients with irritable bowel syndrome (IBS), and associated with inadequate adherence. We aimed to examine the efficacy of an individualized-selective dietary intervention, based on Leukocyte activation to food-components (i.e. Alcat-guided diet) in IBS.

**Methods:**

A randomized, double-blind, sham-controlled trial allocated IBS patients at Sheba Medical Center (Ramat Gan, Israel) between 5 August 2020 and 11 April 2022 to either an Alcat-guided or a sham-balanced diet for 8-week treatment. The primary outcome was a 50-point reduction on the IBS-symptom severity scale (IBS-SSS). The rates of improvement in the IBS Global Improvement Scale (IBS-GIS) and positive response (yes/no) were defined as secondary and exploratory outcomes, respectively.

**Results:**

A total of 68 patients with IBS-D/M (44/24) were enrolled. Baseline characteristics were comparable between groups except for a higher median IBS-SSS score in the Alcat group compared with controls [390 (305–435) vs 330 (240–390), *P *= 0.013]. At week 8, 30 of 35 (85.7%) patients in the Alcat group met the primary outcome, compared with 18 of 33 (54.5%) of controls (*P = *0.005). Alcat patients had higher rates of IBS-GIS improvement (74.3% vs 42.4%, *P = *0.008) and positive response (85.7% vs 57.6%, *P = *0.010) compared with controls at week 8. Among a balanced sub-cohort of patients (*n *= 49) with IBS-SSS score of 250–450 [median: 380 (305–410) vs 355 (310–400) for the Alcat and control groups, respectively, *P = *0.487], the primary outcome was still more commonly achieved among Alcat patients compared with controls (90% vs 59%, *P = *0.022). No serious adverse events were reported.

**Conclusion:**

Immune-based personalized-diet was more efficient than sham-based diet for reducing symptoms in IBS patients, and may serve as a safe treatment option in this population.

## Introduction

Most patients with irritable bowel syndrome (IBS) report specific food-related symptoms [1–3] which may involve food allergy (mainly non-IgE mediated [4, 5]), local immune activation (IgE and mast cell-mediated [6]), and food intolerance [7, 8]. Two endoscopic methods have been shown to detect food item-triggered mucosal changes in susceptible patients using either confocal laser endomicroscopy [5, 9] or the colonoscopic allergen-provocation test [10]. However, these tests are invasive, expensive, have imperfect performance [9, 11], and are not included in current IBS guidelines, which endorse dietary counseling to all IBS patients, including a low FODMAPs diet as a therapeutic option [12–14]. However, these non-personalized approaches are challenging for patients and are associated with significant rates of non-adherence [8, 13].

Non-invasive methods to individualize dietary intervention among patients with IBS have been widely investigated. Among these, an IgG food antigen-based diet has shown several advantages [15–17], but it is still controversial and remains largely experimental [18]. The leukocyte activation test (Alcat-based diet) yields individualized elimination diets based on food-induced morphological modifications of peripheral blood leukocytes. This method was introduced in a previous short-term randomized controlled trial with a relatively small-size IBS cohort [19]. We aimed to examine the clinical efficacy of an Alcat-guided dietary intervention for patients with IBS-diarrhea predominant/mixed habits (IBS-D/M) compared with a sham-based diet.

## Material and methods

This randomized, double-blind, two-arm trial compared the efficacy of an 8-week Alcat-based personalized diet versus a sham-balanced diet for treating IBS. The study was conducted at Sheba Medical Center (Ramat Gan, Israel) between August 2020 and April 2022. The protocol was developed by a board of experts, composed of gastroenterologists including specialists in nutrition and neuro-gastroenterology, registered dieticians, a nurse, and a research coordinator. Patients were recruited from our outpatient gastroenterology clinic, external gastroenterology, and primary care clinics, and through a call published on local physician networks. The trial was registered on ClinicalTrial.gov NCT05616429.

Notably, registration of the study on ClinicalTrials.gov (13 November 2022) was completed after study initiation (5 August 2020) due to administrative delays related to the COVID-19 pandemic. Importantly, all study outcomes were predefined prior to any access to or inspection of raw study data, and all analyses were subsequently conducted independently and without bias.

### Eligibility and exclusion criteria

Adult patients diagnosed with IBS-D/M [20] were eligible for this trial. Only those with active disease [i.e. IBS Symptom Severity Scale (IBS-SSS) >150] [19, 21] were screened. Patients with IBS-C, other gut disorders, or recent antibiotic use (within 1 month) were excluded (see Supplementary Material for details). Only patients with IBS without predominant constipation were included, based on evidence suggesting that diarrhea-predominant and mixed IBS phenotypes are more strongly associated with increased intestinal permeability and immune-inflammatory activation, mechanisms hypothesized to be relevant to leukocyte-based food sensitivity testing [22–24]. Patients were recruited between 5 August 2020 and 11 April 2022.

### Data extraction

At baseline, the following demographic and clinical characteristics were gathered: age, sex, body mass index, IBS type, personal/family history of atopy, family history of IBS, smoking, and previous diet intervention. Blood from each patient was drawn for C-reactive protein (CRP) and soluble syndecan-1 (SDC-1). Stool samples were collected for fecal calprotectin (FC).

### Treatment allocation and randomization

Patients were randomly assigned in a 1:1 ratio to receive either the experimental diet (Alcat-based diet group) or the alternative diet (sham-based diet group) in an 8-week trial. The allocation sequence was generated using a computerized random sequence generator within a web-based electronic case report form, stratified by sex and IBS subtype. Patients, investigators, and the dietitian were blinded to study-group allocation (double-blind design). Specifically, the dietitian counseling patients regarding foods to avoid was unaware whether the exclusion list provided was based on Alcat testing or generated for the sham condition.

### Interventions

The Alcat-based diet was determined by leukocyte activation in response to 200 food samples (details provided in the Supplementary Material and Supplementary Fig. S1). Sham diet reports were presented in an identical format to Alcat results (Supplementary Fig. S2), with foods designated as “reactive” selected at random from a predefined food list using a computerized algorithm. To minimize potential bias, the sham food selection was not guided by known IBS trigger profiles or patient characteristics and was generated independently of clinical data. Commonly reactive foods were not preferentially included or excluded in either group, and the number and distribution of excluded food items were comparable between the Alcat-based and sham-based diet reports. Thus, dietary recommendations in the Sham group were intentionally arbitrary and non-personalized, serving as a control condition rather than a structured elimination diet. A non-blinded research coordinator, not involved in patient management or outcome assessment, provided the coded dietary reports to the blinded dietitian. Patients in both groups were instructed to avoid the foods listed in their respective reports for the duration of the intervention.

### Study follow-up and questionnaire assessments

All patients were monitored by physicians and dieticians, receiving diet instructions and supervision throughout the 8-week follow-up. At baseline, patients completed the Rome-IV IBS diagnostic questionnaire, which a physician reviewed to confirm eligibility for IBS-D/IBS-M. At both the first visit (study initiation) and 8-week follow-up, patients completed the IBS-SSS, IBS Quality of Life questionnaire (IBS-QoL) [21], a visual analogue scale for IBS (VAS-IBS) [25, 26] with seven scales (abdominal pain, diarrhea, constipation, bloating and flatulence, vomiting and nausea, influence on well being, and intestinal symptoms’ effect on daily life), and the Bristol Stool Form Scale [26].

At the 4- and 8-week timepoints, patients completed the global assessment of improvement questionnaire (IBS-GAI) [27, 28]. At 8 weeks, they also completed the global improvement scale (IBS-GIS) [29], adequate relief (IBS-AR) [28], and a positive response (yes/no). Moderate/substantial improvement was defined as a significant response in the IBS-GIS or the IBS-GAI scoring systems [27, 29, 30].

Dietary intake (i.e. types and amounts of consumed foods) during follow-up was assessed using self-reported 3-day food records, as previously described [31]. Adherence to the assigned diet was evaluated at the 4- and 8-week follow-up visits and categorized as non-adherence (< 50%), partial adherence (50%–80%), or complete adherence (> 80%), based on the reported frequency of consumption of foods listed as reactive. All dietary records were routinely reviewed during scheduled follow-up visits by a registered dietitian, who assessed completeness and consistency of the reports and discussed adherence with participants. No objective biomarkers (e.g. caloric intake measurements or weight stability) were used to validate dietary adherence.

### Laboratory tests

At baseline and at 8 weeks, sera were tested for CRP and SDC-1, a potential marker of mucosal permeability [32], and stools for FC (technical details are provided in the Supplementary Material). Laboratory technicians were blinded to any clinical information during follow-up.

### Study outcomes

Patients in the Alcat group were compared for the pre-defined outcomes with the patients in the Sham group. The primary outcome was defined as a 50-point reduction in the IBS-SSS at the 8-week timepoint compared with baseline, as this change has been proven to adequately reflect symptomatic improvement in IBS [21]. The rates of IBS-GAI significant response at 4 and 8 weeks, IBS-GIS significant response at 8 weeks, IBS-AR positive response at 8 weeks (i.e. a significant improvement on daily life) were defined as secondary outcomes, as well as within-groups and between-groups IBS-SSS, IBS-QoL, VAS-IBS, SDC-1, and FC level changes during follow-up. The rate of a positive response to the diet at 8 weeks (an overall improvement) was defined as an exploratory outcome. All adverse events were monitored and recorded during the regularly scheduled visits.

### Sample size

A precise power sample calculation was not feasible as a previous study using the same Alcat assay did not report the proportion of patients achieving a pre-defined cutoff for symptomatic improvement, but rather reported the mean improvement of the score in the group as a whole, using a different score—the IBS-GIS score [19]. Therefore, we pragmatically estimated a sample size of 60 patients (30 per treatment arm), which was also in line with a previously reported food provocation study [5] and other placebo trials in IBS [33]. Assuming a 10%-dropout rate, we planned to enroll 70 patients in this pilot trial, representing a higher number of patients compared with the previous study that tested the Alcat-guided diet (i.e. 58 patients) [19].

### Statistical analysis

Data analyses commenced after all study assessments were completed. Categorical variables were presented as proportions. Continuous variables were expressed as mean ± standard deviation (SD) if normally distributed by the Shapiro–Wilk test, otherwise as median and interquartile range (IQR). Comparisons between the study arms were performed using the two-sample *t*-test or Mann–Whitney *U*-test for normally and non-normally distributed continuous variables, respectively. Within-group pre-post comparisons were performed by the paired *t*-test or Wilcoxon signed-ranks test for normally and non-normally distributed data, respectively.

The χ^2^ test with Yates correction was used for comparisons of categorical variables, where indicated. Given the exploratory design, emphasis was placed on effect size estimation and confidence interval (CI) rather than *post hoc* power calculations. Effect sizes were reported as odds ratios (ORs) for categorical outcomes, while the effect size of continuous outcomes was calculated by the following formula: (Z value of the Wilcoxon signed-rank test)/√N, where N was the sample size. Non-responder imputation was employed in dichotomous efficacy analyses.

In order to address baseline imbalances in IBS-SSS, smoking status, and baseline SDC-1 levels between the study groups, we performed an analysis of covariance (ANCOVA), with treatment allocation as a fixed factor and baseline IBS-SSS included as a covariate. Smoking status was additionally included as a covariate due to baseline imbalance and its potential clinical relevance. To assess potential effect modification, an initial ANCOVA model included an interaction term between treatment allocation and baseline IBS-SSS. As no statistically significant interaction was observed, the interaction term was not retained in the final model, and results are therefore presented from a parsimonious main-effects ANCOVA.

Notably, incorporation of baseline SDC-1 levels as a covariate resulted in a meaningful reduction in the analyzable sample size, as this measure was missing for 12 participants, and it did not show a significant association with IBS-SSS at 8 weeks. Therefore, SDC-1 was omitted from the final model.

Because continuous follow-up IBS-SSS scores were unavailable for a subset of participants from the Sham group only due to differential dropout (*n *= 6), the ANCOVA of continuous outcomes was conducted as a per-protocol analysis including participants with complete baseline and 8-week IBS-SSS data. Estimated marginal means with 95% CI were derived from the ANCOVA models, and pairwise group comparisons were performed using Bonferroni adjustment to account for multiple comparisons. Distributional properties of IBS-SSS at week 8 were examined and showed no substantial deviation from normality (skewness -0.13, kurtosis -1.03), supporting the use of ANCOVA.

Binary outcomes, including the primary endpoint, were analyzed according to the intention-to-treat principle, with non-responder imputation applied to participants who discontinued the study. Continuous outcomes were analyzed using a per-protocol approach, restricted to participants with complete baseline and 8-week measurements, as follow-up raw scores were unavailable for those who discontinued the study. Accordingly, sample sizes differ between intention-to-treat and per-protocol analyses.

Subgroup sensitivity analyses, included comparisons of the primary outcome rates were conducted in distinct populations, such as patients with a personal/family history of atopy, a family history of IBS, or a previous dietary intervention.

All statistical tests were two-sided, and *P *< 0.05 was considered as statistically significant. Statistical analyses were conducted using SPSS software (IBM SPSS Statistics for Windows, version 26, IBM, Armonk, NY, USA).

### Ethics approval and consent to participate

This study was carried out in accordance with the ethical guidelines of the Declaration of Helsinki. It was approved by the Sheba Medical Center ethics committee (Helsinki protocol SMC-20-6834, 26 May 2020). Written informed consent was obtained from all patients.

## Results

### Patients’ baseline characteristics

**Figure 1:**
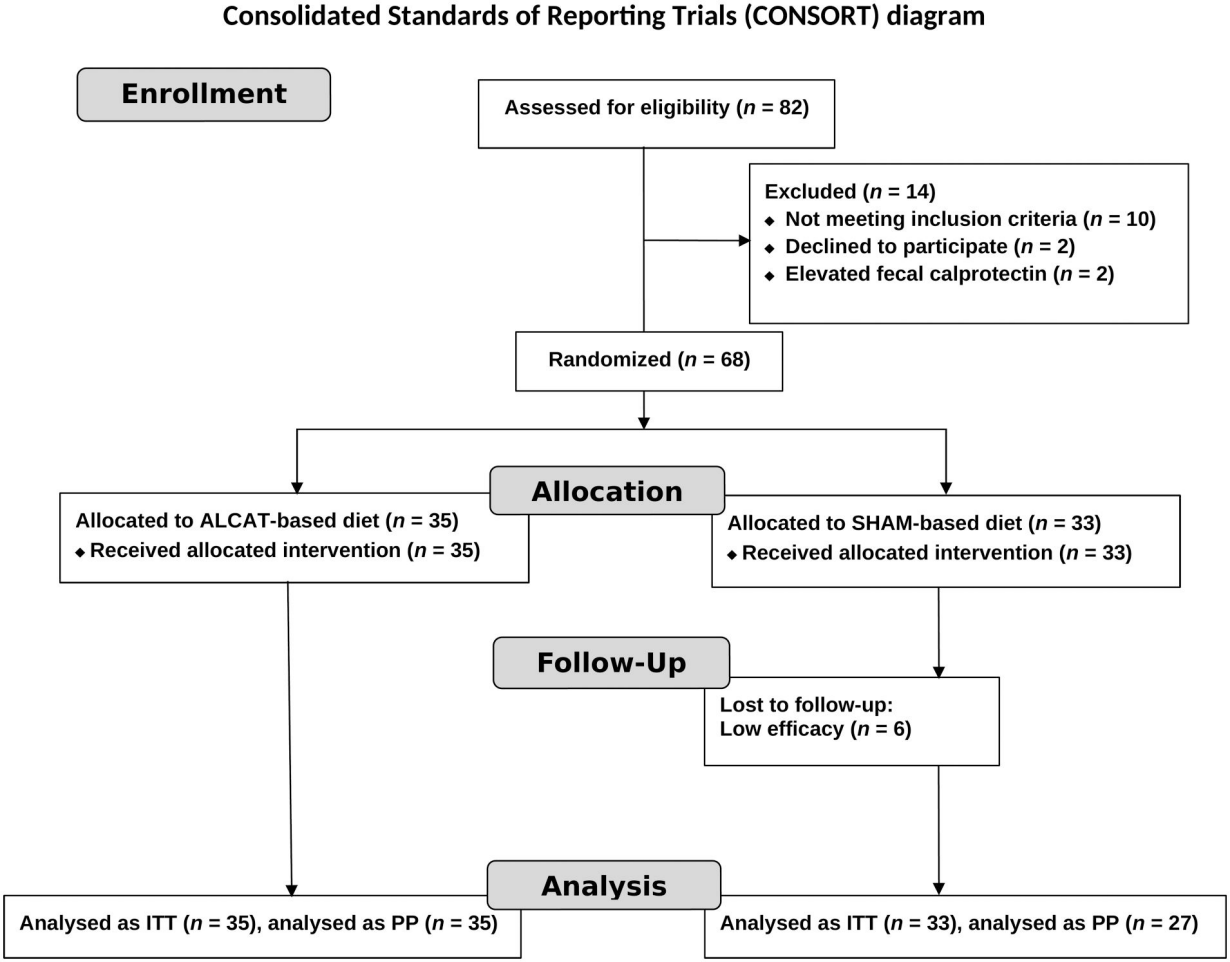
Consolidated Standards of Reporting Trials (CONSORT) diagram of this study. ITT, intention to treat analysis.

Of 82 patients who were screened 12 were excluded because they did not fulfill the ROME-IV criteria for IBS-D/M (*n *= 10), or they did not agree to adhere to the elimination diet (*n *= 2). Two patients were excluded because of elevated FC levels at baseline. In all, 68 patients were enrolled and allocated to the Alcat (*n *= 35) or Sham (*n *= 33) groups (Fig. 1).

Demographic variables were comparable between the groups (Table 1). Most patients were diagnosed with IBS-D (68.6% in Alcat group vs 60.6% in controls, *P *= 0.492), while a minority had IBS-M Data on chronic medications are provided in Supplementary Table S1.

**Table 1:** Patient’s baseline characteristics

Variable	Alcat group	Control group	*P*-value
	(*n *= 35)	(*n *= 33)	
Age, years	38 (27–47)	32 (28–39)	0.387
Male, *n* (%)	13 (37.1)	9 (27.3)	0.385
Body mass index, kg/m^2^	25.15 (21.36–27.61)	22.20 (20.06–25.70)	0.134
IBS type by Rome criteria			0.492
Predominant diarrhea (IBS-D)	24 (68.6)	20 (60.6)	
Mixed bowel movements (IBS-M)	11 (31.4)	13 (39.4)	
Personal and/or family history of atopic disorder	19 (54.3)	15 (45.5)	0.393
Family history of IBS	14 (40.0)	16 (48.5)	0.548
Smoking	3 (8.6)	10 (30.3)	0.023
Previous diet intervention	22 (62.9)	14 (42.4)	0.092
Clinical response to previous diet intervention	10/22 (45.5)	8/14 (57.1)	0.494
IBS questionnaire scores			
IBS-SSS	390 (305–435)	330 (240–390)	0.013
IBS-QoL	105 (85–126)	97 (76–125)	0.351
Stool consistency (Bristol stool form scale)	6 (5–7)	6 (5–7)	0.985
VAS-IBS scores			
Abdominal pain	80 (65–100)	80 (70–90)	0.367
Diarrhea	80 (80–100)	80 (70–100)	0.532
Constipation	0 (0–65)	20 (0–50)	0.413
Bloating	90 (70–100)	70 (50–100)	0.314
Intestinal symptoms’ effect on daily life	80 (60–95)	80 (60–100)	0.901
Nausea/vomiting	20 (0–70)	30 (0–70)	0.716
Influence on well being	90 (80–100)	85 (70–100)	0.766
Laboratory measures			
Serum hemoglobin	13.8 (12.6–14.8)	13.4 (12.9–14.4)	0.457
Fecal calprotectin	40 (30–88)	35 (30–86)	0.754
Soluble syndecan-1	32.4 ± 11.5	27.2 ± 6.7	0.047
C-reactive protein	0.52 (0.5–2.3)	0.5 (0.5–1.3)	0.305

IBS, irritable bowel syndrome; IBS-SSS, IBS Symptom Severity Scale; IBS-QoL, IBS Quality of Life questionnaire; VAS-IBS, visual analogue scale for IBS.

A previous attempt at dietary intervention was more common in the Alcat group compared with controls (62.9% vs 42.4%, *P *= 0.092), with equal response rates (45.5% vs 57.1%, *P *= 0.494). Baseline questionnaire and Bristol Stool Form Scale scores were comparable between the groups, except for a higher baseline IBS-SSS in the Alcat compared with the Sham group [median of 390 (305–435) vs 330 (240–390), *P *= 0.013]. While the mean SDC-1 level was higher in the Alcat group compared with the Sham group (32.4 ± 11.5 vs 27.2 ± 6.7, *P *= 0.047), no difference was observed between the groups in CRP and FC levels (Table 1).

### Study outcomes

**Figure 2:**
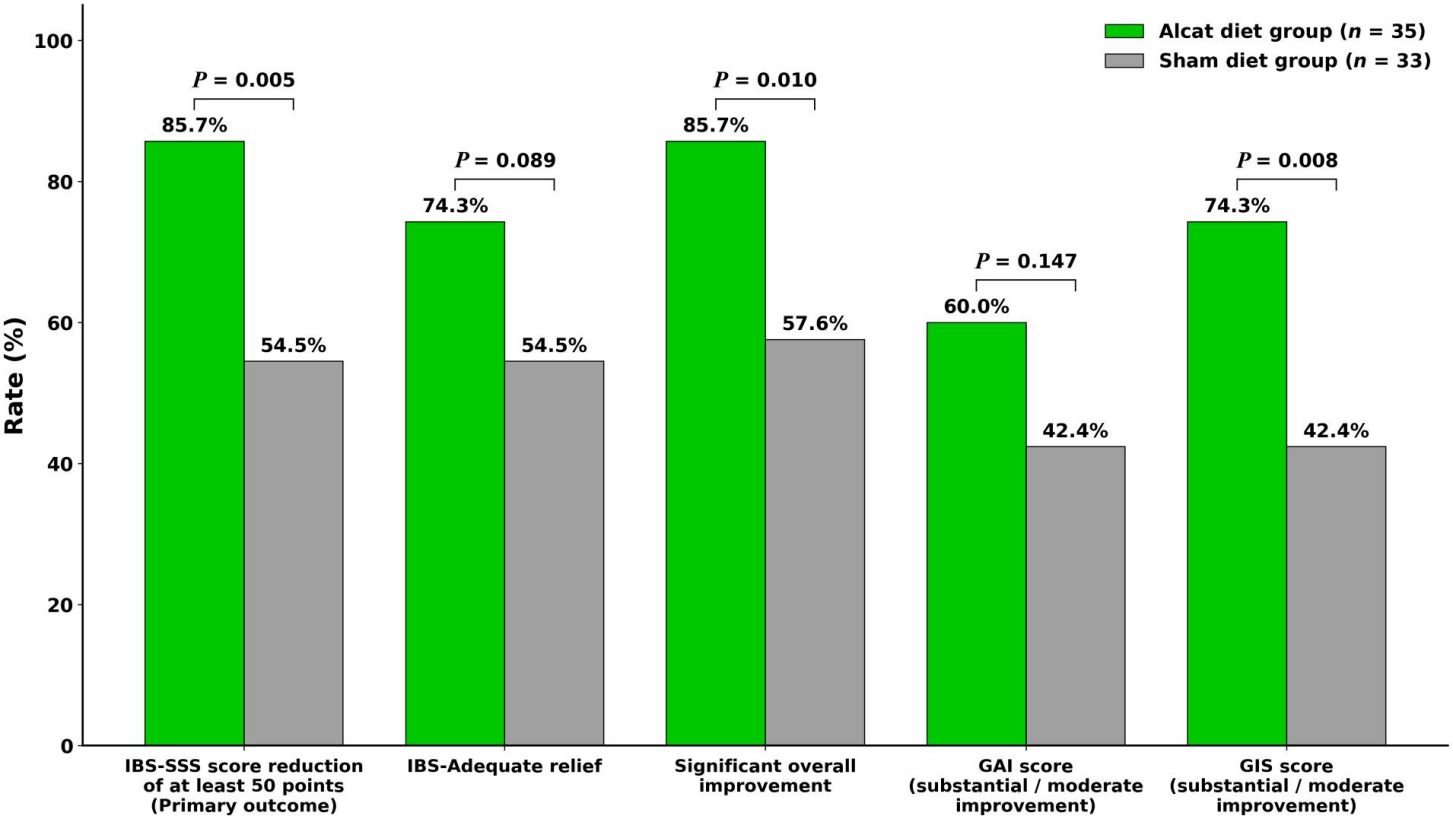
Study outcome rates at the end of the 8-week follow-up in the Alcat-based diet and the Sham-based diet groups. IBS, irritable bowel syndrome; IBS-SSS, IBS Symptom Severity Scale; GAI, global assessment of improvement; GIS, global improvement scale.

In the Alcat group, 30 of 35 (85.7%) patients met the primary outcome compared with 18 of 33 (54.5%) patients in the group (OR 5.000, 95% CI 1.554–16.089, *P *= 0.005). As shown in Fig. 2, more patients in the Alcat group experienced an improvement in IBS-GIS compared with the Sham group (74.3% vs 42.4%, respectively, OR 3.921, 95% CI 1.406–10.930, *P *= 0.008). The IBS-AR score showed a higher rate of improvement with a significant influence on daily life in the Alcat compared with the Sham group (74.3% vs 54.5%, OR 2.407, 95% CI 0.867–6.68, *P *= 0.089). For the exploratory outcome, higher proportions of positive responses were reported in the Alcat compared with the Sham group (85.7% vs 57.6%, OR 4.421, 95% CI 1.37–14.27, *P *= 0.010).

**Figure 3:**
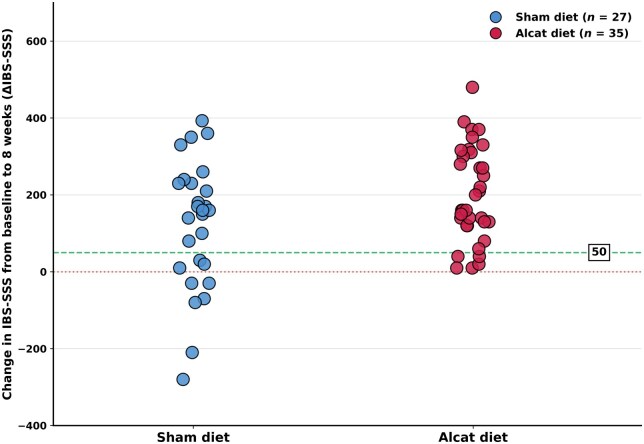
Individual changes in IBS Symptom Severity Score (IBS-SSS) from baseline to 8 weeks by treatment group.

Between-group comparisons of pre-post score changes during follow-up demonstrated a significant difference in favor of the Alcat compared with the Sham group in IBS-SSS (mean change: 201 ± 122 vs 121 ± 167, *P *= 0.034), the abdominal pain component of the VAS-IBS (mean change: 49.71 ± 29.85 vs 31.67 ± 28.85, *P *= 0.020), and with a non-significant improvement in the diarrhea component of the VAS-IBS (*P *= 0.072) and the IBS-QoL (*P *= 0.070). No difference was observed in either FC or SDC-1 levels between groups during follow-up (Table 2). Individual changes in IBS-SSS from baseline to 8 weeks by treatment group are illustrated in Fig. 3.

**Table 2:** Within-groups and between-groups comparisons in regard irritable bowel syndrome (IBS) scoring systems and laboratory measures over time (Δ score change during follow-up) among patients in the Alcat group and the control group

Variable	Within-group comparisons		Between-groups comparisons		*P*-value^a^	Effect^a^ size	*P*-value^b^	Effect size^b^	*P*-value^c^
	Alcat group	Control group	Alcat group	Control group					
	(*n *= 35)	(*n *= 27)	(*n *= 35)	(*n *= 27)					
IBS-SSS					<0.001	0.61	0.002	0.43	0.034
Baseline	390 (305–435)	330 (240–390)	201 ± 122	121 ± 167					
8 weeks	130 (91–250)	150 (95–320)	–	–					
IBS-QoL					<0.001	0.49	0.024	0.35	0.070
Baseline	105 (85–126)	99 (80–125)	22 (9.5–37)	6 (-5 to -29.5)					
8 weeks	75 (58–96)	76 (62–96)	–	–					
Stool consistency (Bristol stool form scale)					<0.001	0.56	<0.001	0.49	0.168
Baseline	6 (5–7)	6 (5–7)	2 (1–3)	1 (0–2.5)					
8 weeks	4 (3–4)	4 (4–5)	–	–					
VAS-IBS score									
Abdominal pain					<0.001	0.60	<0.001	0.54	0.020
Baseline	20 (0–35)	20 (10–30)	49.71 ± 29.85	31.67 ± 28.85					
8 weeks	80 (50–100)	50 (30–80)	–	–					
Diarrhea					<0.001	0.59	<0.001	0.49	0.072
Baseline	20 (0–20)	20 (5–30)	51.00 ± 30.43	34.44 ± 40.86					
8 weeks	80 (45–100)	80 (30–93)	–	–					
Constipation					0.019	0.28	0.399	0.11	0.324
Baseline	100 (35–100)	80 (60–100)	0 (0–20)	0 (-10 to -15)					
8 weeks	100 (80–100)	100 (65–100)	–	–					
Bloating					<0.001	0.52	0.001	0.43	0.43
Baseline	10 (0–30)	20 (0–40)	38.28 ± 36.17	26.48 ± 36.05					
8 weeks	70 (35–95)	50 (25–75)	–	–					
Intestinal symptoms’ effect on daily life					<0.001	0.55	<0.001	0.50	0.54
Baseline	20 (5–40)	20 (5–40)	30 (15–75)	30 (2.5–55)					
8 weeks	80 (40–100)	60 (35–90)	–	–					
Nausea/vomiting					<0.001	0.41	0.017	0.32	0.817
Baseline	80 (30–100)	70 (30–95)	10 (0–45)	10 (0–60)					
8 weeks	100 (100–100)	100 (75–100)	–	–					
Influence on well being					<0.001	0.57	<0.001	0.51	0.425
Baseline	10 (0–20)	15 (0–30)	40 (20–60)	20 (2.5–60)					
8 weeks	60 (30–80)	50 (20–80)	–	–					
Soluble syndecan-1					0.768	–	0.507	–	0.549
Baseline	32.4 ± 11.5	27.4 ± 6.7	0.271 ± 5.07	-0.372 ± 2.76					
8 weeks	32.1 ± 10.1	27.8 ± 7.3	–	–					
Fecal calprotectin					0.223	–	0.588	–	0.866
Baseline	40 (30–88)	35 (30–86)	0 (-9 to 34)	0 (-16 to 35)					
8 weeks	30 (30–72)	36 (30–79)	–	–					

IBS-SSS, IBS Symptom Severity Scale; IBS-QoL, IBS Quality of Life questionnaire; VAS, visual analogue scale for IBS.

a Alcat group (within-group comparison).

b Control group (within-group comparison).

c Between-groups comparisons.

Within-group differences over time were observed in both groups for most scales. Thus, improvement was demonstrated among most patients during follow-up, independent of the trial intervention. Yet, no difference was observed within groups regarding FC and SDC-1 levels during follow-up (Table 2, and Supplementary Figs S3 and S4).

In the per-protocol analysis of the continuous outcome (IBS-SSS), a multivariable ANCOVA adjusting for baseline IBS-SSS and smoking status did not demonstrate a statistically significant independent effect of the intervention on IBS-SSS at 8 weeks. Importantly, baseline IBS-SSS was not independently associated with follow-up symptom severity (*P *= 0.202), indicating that initial symptom burden did not drive final outcomes. Adjusted mean IBS-SSS scores were consistently lower in Alcat-based diet group compared with the control group [β coefficient -22.1 (-92.1 to 47.8), *P *= 0.529], although this difference did not reach statistical significance. Smoking status showed a borderline association with higher IBS-SSS scores at follow-up [β coefficient 79.0 (-8.0 to 166.1), *P *= 0.074], suggesting poorer symptomatic improvement among smokers (Table 3).

**Table 3:** Multivariable ANCOVA model for IBS-SSS at 8 weeks

Variable	β coefficient (95% confidence interval)	*P*-value
Intervention (Alcat vs sham)	-22.1 (-92.1 to 47.8)	0.529
Baseline IBS-SSS (per 10-point increase)	2.5 (-1.4 to 6.3)	0.202
Smoking (yes vs no)	79.0 (-8.0 to 166.1)	0.074

### Diet composition and adherence rates

**Figure 4:**
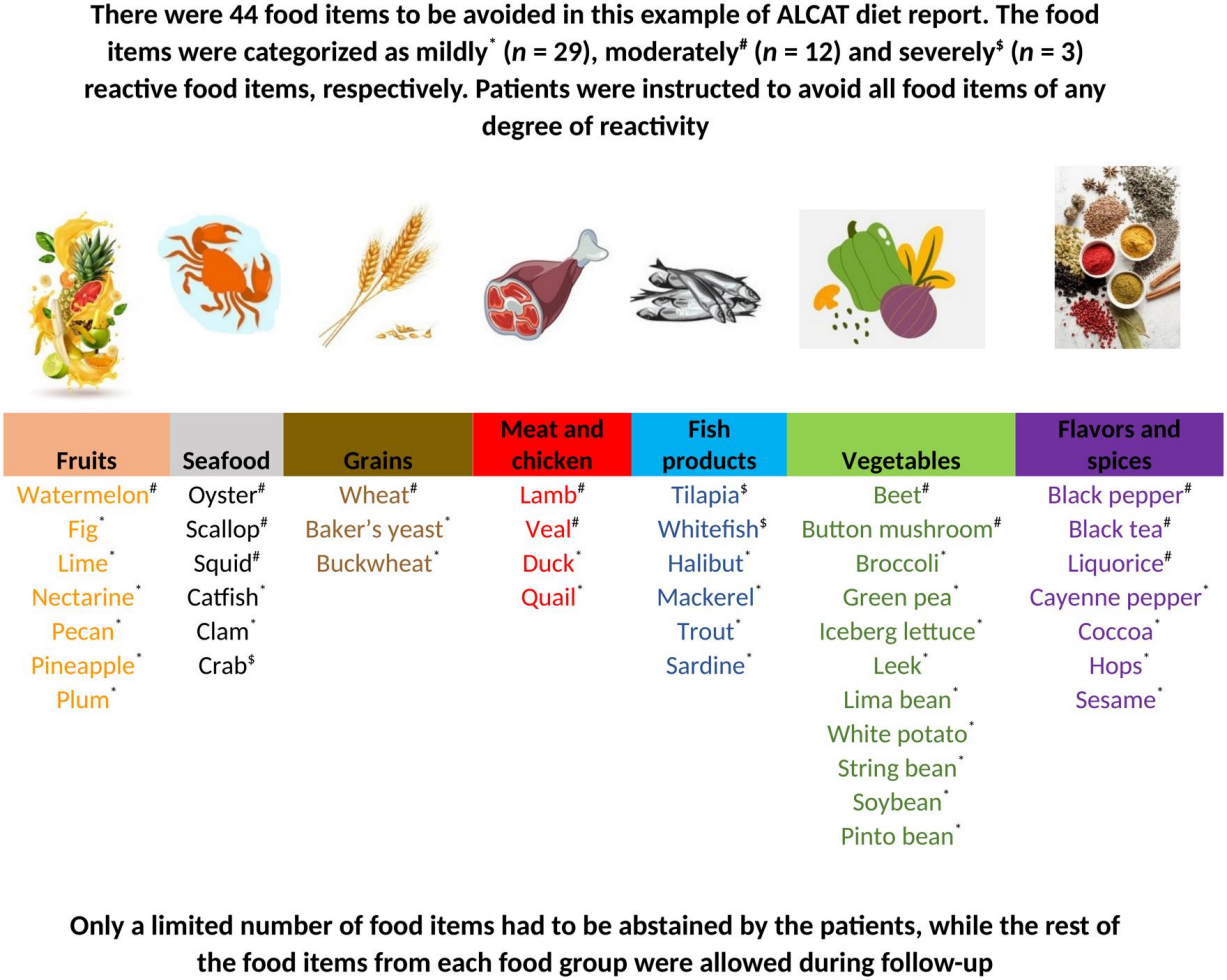
An example of the forbidden food item list, based on an Alcat-diet report.

The median number of food items to avoid was similar in both groups (Alcat: 50, 95% CI 46–53; Sham: 52, 95% CI 44–54, *P *= 0.909). These comprised specific items from limited food groups, with other items in those groups allowed (Fig. 4). Table 4 lists the top 30 foods to avoid in each group.

**Table 4:** The most common food items (i.e. top-30, divided into the main food groups), which patients in the study groups were instructed to avoid of during follow-up, based on the Alcat-diet and the randomly selected sham-diet reports

Alcat-diet group		Sham-diet group	
Mussel	Saffron	Octopus	Cucumber
Oyster	Malt	Whitefish	Romaine lettuce
Crab	Romaine lettuce	Rye	Parsnip
Squid	Turnip	Peanut	Iceberg lettuce
Whitefish	Acorn squash	Sheep’s milk	Eggplant
Sardine	Radish	Millet	Plum
Trout	Swiss chard	Black tea	Mango
Tilapia	White potato	Corn	Orange
Walnut	Beet	Leek	Pineapple
Wheat	Zucchini squash	Pumpkin	Strawberry
Venison	Apricot	Radish	Cantaloupe
Veal	Pomegranate	Yellow squash	Nectarine
Honey	Nectarine	Turnip	Pomegranate
Hops	Grape	Zucchini squash	Kiwi
Black tea	Watermelon	Green pea	Watermelon

Patients in Alcat group reported higher diet adherence than those in Sham group at 4 and 8 weeks (see Supplementary Table S2 for details).

### Sensitivity analysis

Among diet-naïve patients, the Alcat-based diet group showed better response than the sham-based diet group (ΔIBS-SSS ≥ 50 rate: 100% vs 57%, *P *= 0.010). Patients with prior dietary intervention experience showed no difference in primary outcome rates between groups (*P *= 0.148). Results of the other subgroup analyses (i.e. personal/family history of atopy/a family history of IBS) are provided in the Supplementary Table S3.

An analysis was conducted in the sub-groups of patients with baseline IBS-SSS scores of 250–450 [median: 380 (305–410) in Alcat group (*n *= 27) vs 355 (310–400) in Sham group (*n *= 22); *P *= 0.487]. As in the main analysis, in this balanced sub-cohort comparison, the primary outcome was achieved more in the Alcat group than in the sham-diet group (90% vs 59%, *P *= 0.022).

### Adverse events

There were no reports of adverse events or serious adverse events in either group.

## Discussion

In this randomized, sham-controlled trial, a higher proportion of IBS-D/M patients who received the Alcat-based diet achieved a ≥ 50-point reduction in the IBS-SSS, compared with the sham-based diet at the 8-week timepoint. Improvements in the IBS-GIS and the abdominal pain component of the VAS-IBS were more commonly achieved in the Alcat group than the Sham group.

Several findings from the present analysis consistently support a beneficial effect of the dietary intervention. First, baseline IBS-SSS was not an independent predictor of symptom severity at 8 weeks, suggesting that treatment outcomes were not merely a function of initial disease severity. This observation strengthens the interpretation that post-intervention differences reflect treatment-related effects rather than regression to the mean. Second, although the ANCOVA analysis did not reach statistical significance, adjusted IBS-SSS scores were consistently lower in the intervention group across all models, indicating a directional effect favoring the intervention. The absence of statistical significance in this context is likely attributable to limited power inherent to the per-protocol analysis of continuous outcomes. Notably, six participants in the control group withdrew before completing follow-up assessments, whereas no dropouts occurred in the intervention group. As a result, continuous IBS-SSS scores at 8 weeks were unavailable for a substantial proportion of controls, increasing variability and attenuating the detectable between-group difference. This interpretation is supported by primary outcome, in which a clinically meaningful improvement (≥ 50-point reduction in IBS-SSS) was significantly more frequent in the intervention group when analyzed according to the intention-to-treat principle. Unlike the continuous analysis, this approach allowed inclusion of all randomized participants and was therefore less sensitive to missing outcome data, providing a more robust estimate of treatment efficacy.

Smoking emerged as a clinically relevant factor associated with poorer symptomatic outcomes, independent of treatment allocation. This finding underscores the importance of lifestyle factors in modulating IBS symptom trajectories and suggests that smoking status may influence both baseline symptom persistence and response to intervention. This finding is in line with previously published studies indicating that smoking is associated with an increased risk of IBS and greater symptom severity, particularly among patients with the IBS-D type [34–36].

Most IBS patients (63%–84%) report food-related symptoms [1, 2, 37], which negatively affects their quality of life [2]. Low-grade mucosal inflammation, particularly mast cell activation, has been suggested as a contributory factor in the pathogenesis of IBS [6]. Mast cells are highly involved in allergic reactions, wherein IgE mast cell-interactions lead to prompt release of inflammatory mediators [38]. It was postulated that immunologic and enteric nervous system interactions following exposure to dietary antigens, subsequently result in gastrointestinal symptoms in IBS patients. Evidence supporting this hypothesis includes intestinal barrier-perturbation (i.e. immediate breaks, increased intervillous spaces, and increased intraepithelial lymphocytes) detected by the confocal laser endomicroscopy, in response to non-IgE induced food exposure [4, 5]. These changes were associated with the patients’ response to exclusion diets. Conceptually, an immune assay-guided diet may facilitate individualized dietary intervention, increasing its efficacy compared with other interventions.

Somewhat in line with mechanistic hypotheses implicating allergic–inflammatory hypersensitivity reactions in food-induced symptoms in IBS, the Alcat-based diet is designed to individualize dietary intervention based on a morphometric assay of leukocyte activation in response to a broad array of food antigens [19].

However, the specific immune pathways underlying the cellular morphometric changes measured by the Alcat assay were not directly assessed in the present study, representing an important limitation. In particular, no mechanistic correlates such as cytokine profiles, mast cell activation, or other mucosal or systemic immune markers were evaluated, precluding biological validation of the assay’s mechanistic relevance.

Nevertheless, the observed association between Alcat test-guided dietary intervention and clinically meaningful symptomatic improvement suggests that immune-mediated food reactivity may contribute to symptom generation in at least a subset of patients with IBS. Future studies should incorporate detailed immunophenotyping approaches, including assessment of allergic and immunologic mediators (e.g. cytokine signatures, mast cell activity, and innate immune responses), to better elucidate the pathways linking leukocyte activation, food sensitivity, and IBS symptomatology. Such mechanistic validation would be essential to further define the pathophysiologic relevance of the Alcat assay and to strengthen the biological rationale for Alcat-based dietary interventions.

The prevalence of perceived food intolerance among IBS patients ranges 50%–70% [7, 8]. The USA, the British, and European guidelines [12–14] all recommend empirical, non-individualized diet eliminations, including a low FODMAPs diet as a treatment option in IBS. Two previously published RCTs demonstrated no difference in efficacy, when a low FODMAPs diet was compared with other traditional diets for 4 weeks [39, 40]. A recent meta-analysis [41] reported that the superiority of low FODMAP diets over other dietary interventions was mainly due to improved abdominal symptoms rather than diarrheal-related symptoms. Non-individualized interventions have several disadvantages including reduced caloric-intake, micronutrient deficiencies, and incomplete patient adherence [8, 13]. The trend toward improvement in diarrhea-related symptoms in the Alcat group compared with the Sham group, may represent an additional advantage of this personalized intervention approach compared with non-individualized diets [15, 39, 40, 41].

Like Ali *et al.* [19], we observed significant symptomatic improvement in the Alcat group compared with the Sham group. Our study had several advantages over the former: a larger sample size, more detailed baseline data enabling subgroup analysis, and a sham-based diet using a random Alcat report rather than allowing the consumption of severely reactive foods in the former study, which could have biased the results in favor of the Alcat diet. Additionally, we used various validated questionnaires and scoring systems, enhancing the robustness and consistency of our findings.

To date, IgG-based elimination diets, another potential approach, remain experimental. Atkinson *et al.* [15] demonstrated the usefulness of a 3-month IgG-based elimination diet, compared with a sham-based diet, although the benefit was not observed at earlier timepoints. While IgG-based diets have been demonstrated to be effective in recent studies [17, 42], these findings cannot be generalized due to the small sample sizes and the limited number of the examined food items.

Patients naïve to dietary intervention were more likely to benefit from Alcat diet than those who had previously tried such interventions. One might speculate that the latter group had less stringent adherence, and/or increased skepticism toward dietary interventions. Alternatively, it may indicate a subgroup of IBS patients who should be managed with medications rather than dietary intervention.

Although adherence was systematically assessed and generally high across both groups, differential adherence may have contributed to treatment effects and cannot be fully disentangled from the intervention itself. In addition, while the trial was double-blinded at the participant, investigator, and dietitian levels, formal assessment of blinding integrity was not conducted, and partial unblinding at the implementation level represents a limitation.

Notably, a substantial improvement was also observed in the sham-diet group, with 54.5% of participants meeting the primary endpoint. This finding is consistent with existing literature demonstrating a strong placebo response in IBS dietary trials [43, 44]. Network meta-analyses have shown that when dietary interventions are compared with sham-based diets or standard dietary advice, active dietary strategies are often not consistently superior, suggesting that the process of dietary counseling itself may contribute significantly to symptom improvement. Structured follow-up and regular interaction with an experienced dietitian may provide reassurance, increase symptom awareness, and enhance perceived control over symptoms, all of which are known contributors to placebo responses in IBS [43, 44]. These findings underscore the importance of accounting for substantial sham effects when interpreting dietary intervention trials in IBS.

This study has several limitations. One is its modest cohort size. However, our cohort was larger than those in previously reported similar studies. This enabled us to conduct multiple subgroup analyses to better characterize responses to intervention in IBS sub-populations. Although the study was not powered for definitive efficacy testing, the consistency of large effect sizes and narrow confidence intervals for key outcomes supports the robustness and clinical relevance of the observed effects. Second, like most studies in this field, we relied on self-reported questionnaires which limited accuracy and reliability across various disease severity categories. Third, patients were screened for active IBS, defined as IBS-SSS > 150, aligning with a previous study in this field [19], though IBS-SSS > 175 is more commonly used [21]. Nevertheless, only one patient had an IBS-SSS below 175 [21] (i.e. 170) at baseline, while all others met IBS-SSS ≥ 190. Fourth, the sham-based diet was randomized, thereby possibly including “reactive” forbidden food items, which could potentially heighten the observed between-group differences. Finally, the baseline median IBS-SSS score was higher in the Alcat compared with the Sham group, which could have influenced the results, although one would assume these patients with more severe baseline IBS are more difficult to treat and may even strengthen the study findings. Nevertheless, a sensitivity analysis restricted to patients with a comparable baseline IBS-SSS score of 250–450 showed that patients in the Alcat group were still more likely to achieve the primary outcome compared with patients in the Sham group.

## Conclusions

An 8-week dietary intervention using an individualized, noninvasive immune-based elimination diet, was associated with significantly higher symptomatic improvement and adherence rates, compared with a sham-based diet in patients with IBS-D/M Thus, the Alcat-based diet could potentially guide dietary intervention in IBS. Further research, with larger sample sizes, combining clinical and mechanistic components, could strengthen the perception that the use of an Alcat-based diet might be considered as an acceptable, effective and safe intervention in this population.

## Supplementary Material

goag058_Supplementary_Data
