# Supplementary material for: Immune-based personalized elimination diet for the treatment of irritable bowel syndrome: a double-blind randomized sham-controlled study
Source: Gastroenterol Rep (Oxf). 2026 Jun 12;14:goag058. doi: 10.1093/gastro/goag058 (PMC13262740; doi:10.1093/gastro/goag058)
Supplement: goag058_Supplementary_Data [file goag058_supplementary_data.zip › Supplementary_material_final.docx]

**Materials and methods**

**Exclusion criteria**

Patients who met the ROME-IV criteria [1] for IBS-C were excluded. Patients with a previous diagnosis of inflammatory bowel disease, celiac or other inflammatory/gut disorder other than IBS, or active malignancy (i.e., solid or hematological origin) were excluded.

Patients who could not consume orally for any reason, who used non-steroidal anti-inflammatory drugs more than once per month and within four weeks prior to inclusion, who used antibiotics within one month before screening, or who had elevated inflammatory biomarkers [i.e., C-reactive protein (CRP) > 2X upper normal limit, and/or fecal-calprotectin (FC) > 150 mcg/mg] were excluded.

Participants were allowed to continue their IBS medications (e.g., fiber, anti-spasmodic, antidepressants, loperamide, etc.) as long as drug doses were stable within 30 days before enrollment and were instructed not to change medications/their dosages during follow-up.

**Leukocytes activation test** [2, 3]

A single sample of de-identified peripheral venous blood was preserved in 3.8 sodium citrate and was sent for testing at a commercial laboratory (Alcat, Cell Science Systems, GmbH, Germany) via overnight courier. Whole blood was tested and red blood cells were lysed just prior to analysis. Leukocytes were separated from whole blood using a density gradient at a high-speed centrifugation. Subsequently, a neutral buffer and an autologous plasma were added back to the cells, which were then aliquoted into a round-bottomed test or control boreholes containing 200 test food-extracts or neutral buffer, respectively. The above samples were incubated for 15 min, with 100 rpm rotation, at 37°C. Cell counts were performed using the electric sensing zone method (Coulter or impedance method) of particle sizing, distributing each cell into one of 256 size channels, thus creating a size-volume distribution curve for each test or control sample. Size-volume cell population distribution in response to a food item exposure was used to determine food intolerance for each food extract and was categorized as severe, moderate, mild or none (i.e., no reaction) based on the distinctive morphologic pattern of each curve.

**Laboratory tests**

At baseline and at 8-week time-point sera were tested for CRP and Syndecan-1 (SDC-1), (implicated as a marker of mucosal permeability [4, 5]) and stools for FC. SDC-1 concentration was determined using human syndecan-1 enzyme-linked immunosorbent assay (ELISA; Medix Biochemica, Diaclone SAS, Besancon, France), and its levels were reported as ng/mL. Serum CRP concentrations were reported as mg/dL. FC concentrations were reported as μg/mg (Buhlmann Laboratories AG, Basel, Switzerland).

**References**

1. Drossman DA. Functional gastrointestinal disorders: what’s new for Rome IV? *Lancet Gastroenterol Hepatol*. 2016;1(1):6-8.

2. Ali A, Weiss TR, McKee D, *et al.* Efficacy of individualised diets in patients with irritable bowel syndrome: a randomised controlled trial. *BMJ open Gastroenterol*. 2017;4(1):e000164.

3. Garcia-Martinez I, Weiss TR, Yousaf MN, *et al.* A leukocyte activation test identifies food items which induce release of DNA by innate immune peripheral blood leucocytes. *Nutr Metab (Lond)*. 2018;15:26.

4. Qing Q, Zhang S, Chen Y, *et al*. High glucose-induced intestinal epithelial barrier damage is aggravated by syndecan-1 destruction and heparanase overexpression. *J Cell Mol Med*. 2015;19(6):1366-1374.

5. Yablecovitch D, Oren A, Ben-Horin S, *et al*. Soluble Syndecan-1: A Novel Biomarker of Small Bowel Mucosal Damage in Children with Celiac Disease. *Dig Dis Sci*. 2017;62(3):755-760.

**Supplementary table 1.** Baseline medications among the study populations divided to the patients in the Alcat-diet and the Sham-diet groups.

| Variable | Alcat-based diet group  (*n* = 35) | Sham-based diet group  (*n* = 33) |
| --- | --- | --- |
| Oral contraceptives | 5 (14.3) | 3 (9.1) |
| Tricyclic antidepressants | 1 (2.9) | 0 (0) |
| Proton pump inhibitors | 2 (5.7) | 4 (12.1) |
| Selective serotonin reuptake inhibitors | 2 (5.7) | 2 (6.1) |
| Serotonin norepinephrine reuptake inhibitors | 1 (2.9) | 1 (3.0) |
| Bupropion | 1 (2.9) | 0 (0) |
| Glutamine | 1 (2.9) | 0 (0) |
| Cannabis | 2 (5.7) | 1 (3.0) |
| Glucagon-like peptide-1 agonist | 1 (2.9) | 0 (0) |
| Anti spasmodic agents | 2 (5.7) | 1 (3.0) |
| Pregabalin | 0 (0) | 1 (3.0) |

**Supplementary table 2**. Adherence to diet rates in both study arms during follow-up.

| Variable | Alcat-based diet group  (*n* = 35) | Sham-based diet group  (*n* = 33) | *P*-value |
| --- | --- | --- | --- |
| 4-week time-point |  |  |  |
| Complete compliance | 34 (97.1) | 25 (75.8) | 0.009 |
| Partial compliance | 1 (2.9) | 3 (9.1) |  |
| No compliance | - | 5 (15.1) |  |
| 8-week time-point |  |  |  |
| Complete compliance | 31 (88.6) | 26 (78.8) | 0.273 |
| Partial compliance | 4 (11.4) | 1 (3.1) |  |
| No compliance | - | 6 (18.2) |  |

**Supplementary table 3**. The rates of a 50-point reduction in the irritable bowel syndrome-severity scoring system (IBS-SSS) at the 8-week time-point in distinct subgroups of IBS patients.

| Variable |  | Alcat-based diet group | Sham-based diet group | *P*-value |
| --- | --- | --- | --- | --- |
| Naïve to dietary intervention | Yes | 100% | 57% | 0.010 |
|  | No | 77% | 50% | 0.148 |
| Personal and/or family history of atopy | Yes | 89% | 73% | 0.370 |
|  | No | 80% | 38% | 0.033 |
| Family history of IBS | Yes | 100% | 62.5% | 0.019 |
|  | No | 75% | 47% | 0.101 |

**Figure legends**

**Supplementary figure 1.** The Alcat Test analyzes the effect of each tested individual food substance on leukocytes. Cellular immune reactions lead to changes in cell volume and cell number (**A**), which are displayed in histograms using a mathematically computerized algorithm evaluation (**B**). The degree of reaction is calculated and "mirrored" as a snapshot in four reaction degrees: severe, moderate, mild, no reaction (**C**). Adapted with a permission from Alcat, Cell Sciences, Germany.

**Supplementary figure 2.** An example of a formal Alcat diet report.

**Supplementary figure 3.** Median scores of IBS Symptom Severity Scale (IBS-SSS) (A) and the IBS Quality of Life questionnaire (IBS-QoL) (B) at baseline and eight weeks (vertical error bars represent the interquartile range) by study group. The *P*-value in each graph represents the between-groups difference over time.

**Supplementary figure 4.** Median VAS scores for abdominal pain [A], diarrhea [B], and bloating [C], at baseline and eight weeks (vertical error bars represent the intraquartile, by study group. The *P*-value in each graph represents the between-groups difference over time. VAS, visual analogue scale for IBS.
