# Supplementary figures and images for: Immune-based personalized elimination diet for the treatment of irritable bowel syndrome: a double-blind randomized sham-controlled study
Source: Gastroenterol Rep (Oxf). 2026 Jun 12;14:goag058. doi: 10.1093/gastro/goag058 (PMC13262740; doi:10.1093/gastro/goag058)

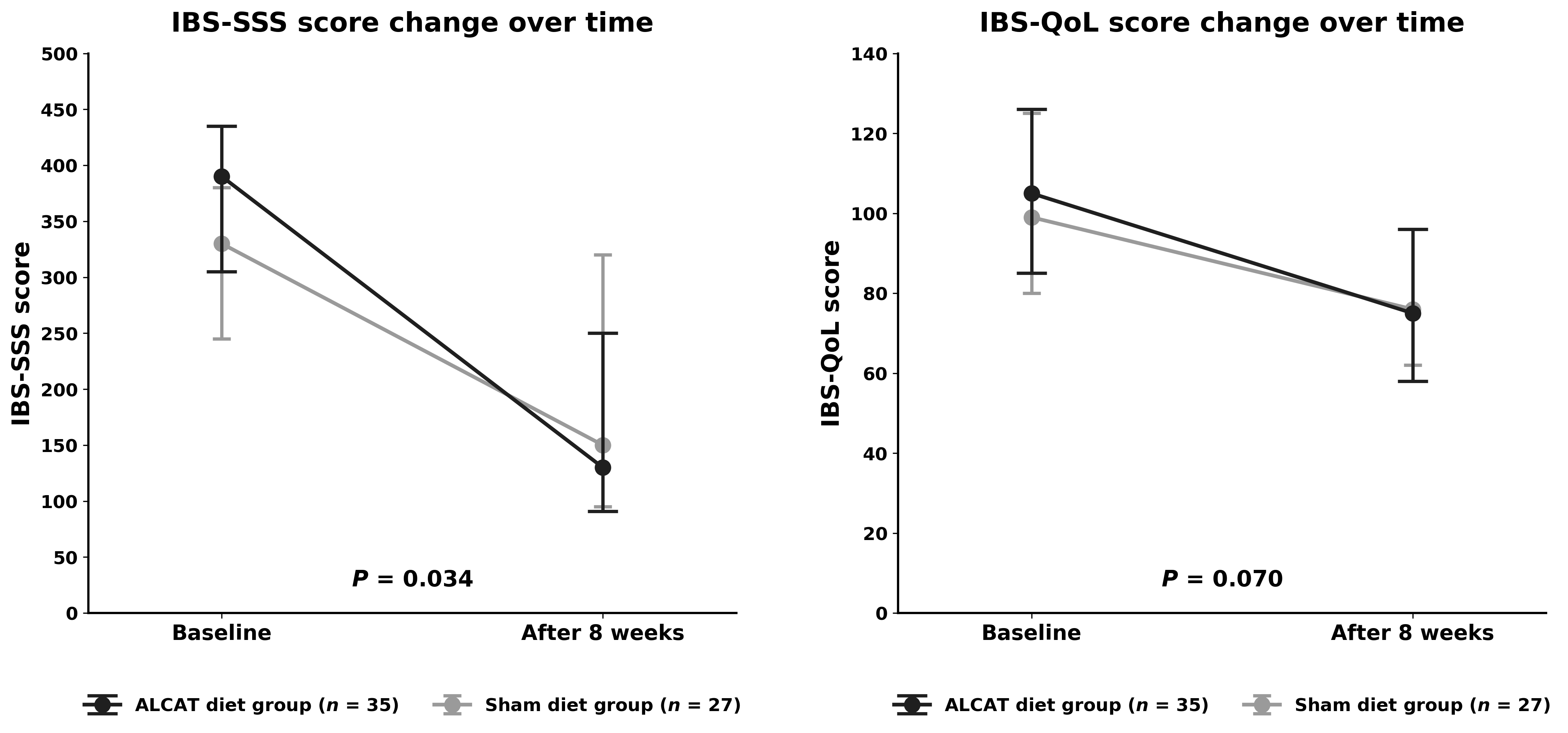

Supplement: goag058_Supplementary_Data [file goag058_supplementary_data.zip › SuppFig3_modified.tif]

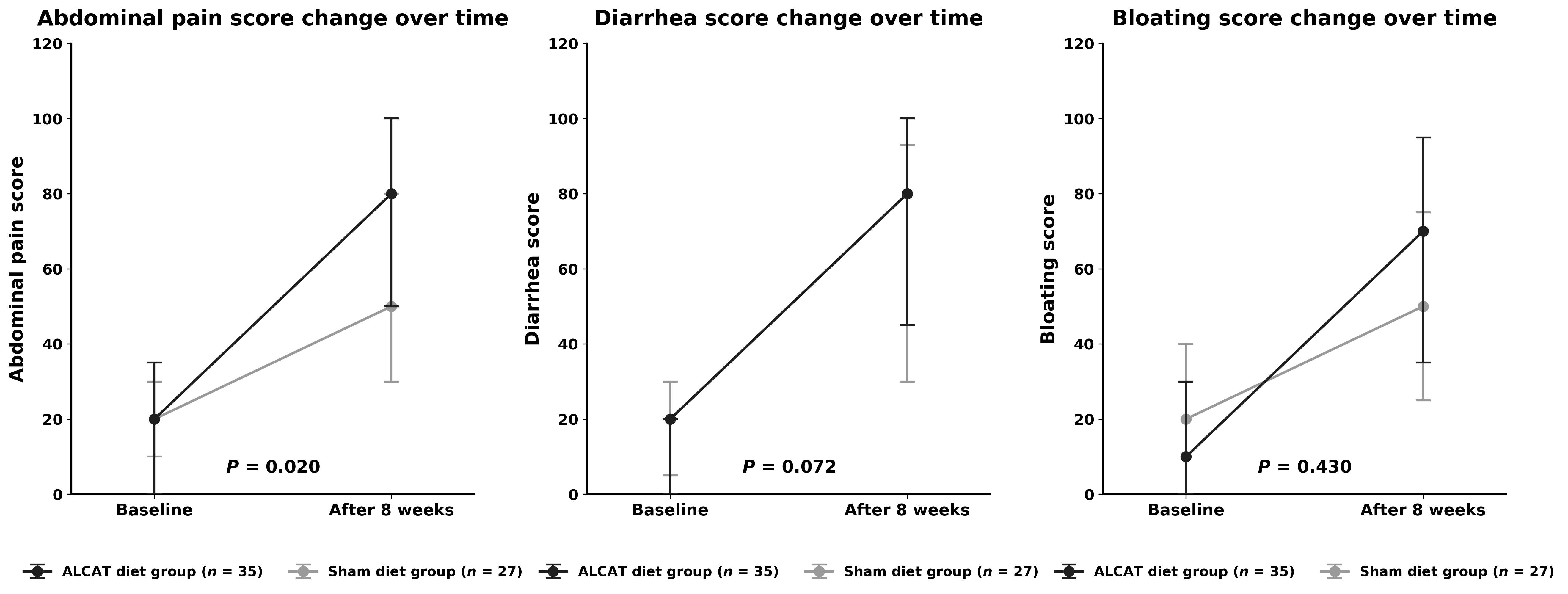

Supplement: goag058_Supplementary_Data [file goag058_supplementary_data.zip › SuppFig4.tif]

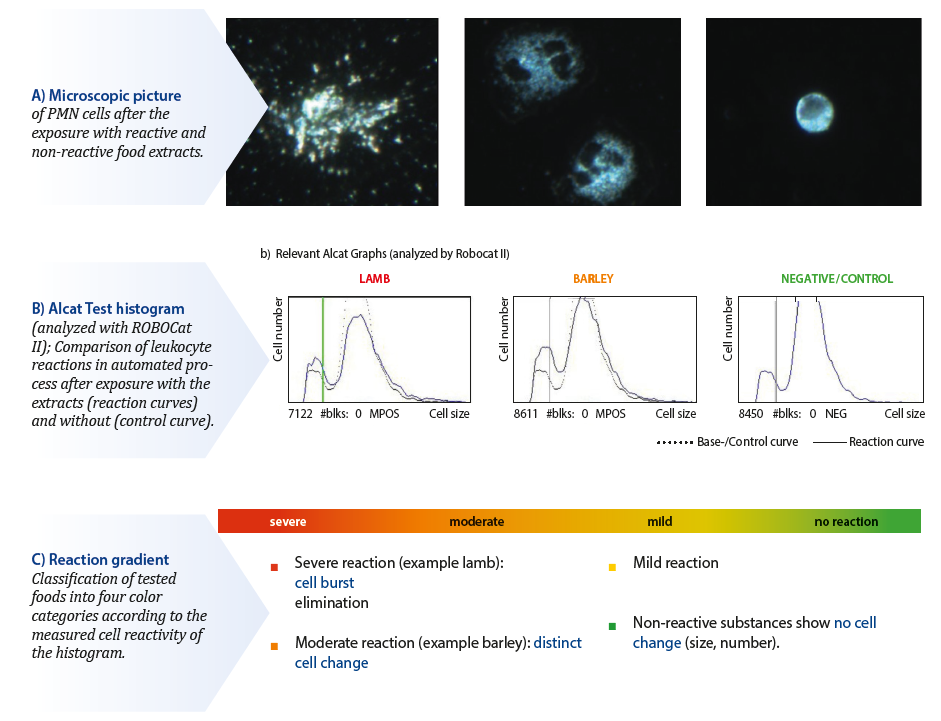

Supplement: goag058_Supplementary_Data [file goag058_supplementary_data.zip › Supp_Fig1_1200.tif]

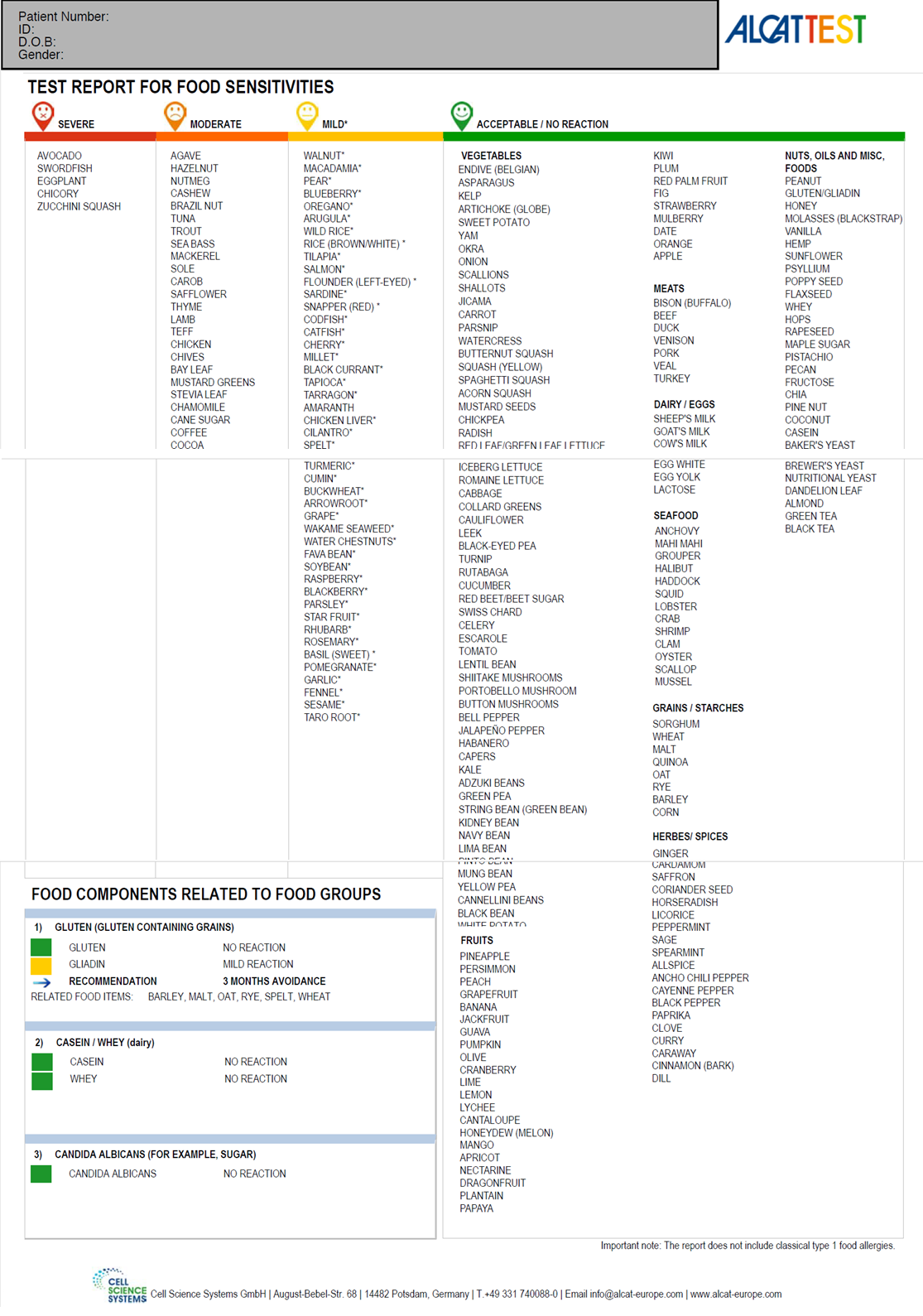

Supplement: goag058_Supplementary_Data [file goag058_supplementary_data.zip › SuppFig2.tif]
